# Fully endoscopic neurosurgery using a two-handed technique for cerebellopontine angle tumors via the retrosigmoid approach

**DOI:** 10.3389/fonc.2024.1485932

**Published:** 2024-12-16

**Authors:** Zhengxing Xie, Yan Zhuang, Jieping Liu

**Affiliations:** ^1^ Department of Neurosurgery, The Affiliated Hospital of Jiangsu University, Zhenjiang, China; ^2^ Neuro-Endoscope and Mini-Invasive Treatment Center, The Affiliated Hospital of Jiangsu University, Zhenjiang, China

**Keywords:** fully endoscopic neurosurgery, cerebellopontine angle, retrosigmoid approach, approaching observation, retractorless technique

## Abstract

**Background:**

Surgery for tumors in the cerebellopontine angle is always a significant challenge due to the densely packed neurovascular structures, the narrow deep location, and the complex relationship between the lesions and surrounding neurovascular structures. Recently, great attention has been given to the neuroendoscope for its exclusive advantages, which have added a new dimension to many classical microscopic surgeries. However, the feasibility and advisability of fully endoscopic neurosurgery for cerebellopontine angle tumors remain to be further evaluated.

**Methods:**

We retrospectively collected the clinical outcomes and endoscopic surgical experience of 12 patients with tumors in the cerebellopontine angle (CPA) from January 2022 to April 2024 in our department. We analyzed patients’ records, radiological neuroimaging, tumor-related variables, surgical procedures, and postoperative outcomes in detail. All patients were regularly followed up with neurological examinations and magnetic resonance imaging (MRI)/computed tomography (CT).

**Results:**

The pathology of the series included five cases of acoustic neuroma, six cases of meningioma, and one case of teratoma. The mean largest diameter of the lesion was 29.5 mm ±8.5 mm. Headache, hearing loss, and dizziness were the top three most common symptoms. All tumors were resected using the hand technique. No hemorrhage, cerebrospinal fluid leaks, or intracranial infections occurred. All patients with meningioma achieved Simpson grade II resection, and the remaining tumors underwent gross total resection, confirmed by both intraoperative and postoperative imaging. Overall, 91.7% of patients maintained normal facial nerve function postoperatively (HB1). One patient with acoustic neuroma experienced transient facial paralysis after surgery (HB2), which resolved during follow-up at 3 months postoperation. Clinical symptoms of all the other patients were resolved or ameliorated after surgery, with no new neurological deficits. The Karnofsky Performance Scale (KPS) scores remained unchanged or improved for all patients postoperatively.

**Conclusions:**

With the accumulation of experience and technological progress, the fully endoscopic retrosigmoid approach could enable safe and effective resection of cerebellopontine angle tumors, providing a panoramic view and illumination of deep-seated structures.

## Introduction

The cerebellopontine angle (CPA) is characterized by densely packed neurovascular structures and narrow, deep operative corridors, which make this area one of the most challenging and demanding regions in the brain. Therefore, surgical excision of tumors in the cerebellopontine angle is extremely challenging (1, 2). Many studies raise concerns regarding surgical mortality and morbidity associated with surgery in the CPA (3). Excessive retraction of the cerebellum and restricted visibility of the surgical field in conventional microscopic surgery are believed to contribute to compromised clinical outcomes. Due to the direct line of light from the microscope, there is always a dilemma between minimal retraction and bright visibility of the deep field in conventional surgery for CPA lesions.

Recently, with the successful implementation of the endoscope in the transnasal approach (4, 5), the improved visualization of the neurovascular structures has gained significant attention from neurosurgeons, leading to the adoption of endoscopically assisted procedures in some neurosurgeries (6–8). Several reports have highlighted the advantages of endoscopic visualization during pituitary adenoma resection (9) and microvascular decompression (10). There also some studies that highlight the application of the endoscope to assist the microscopic resection of skull base tumor (6). However, few reports on fully endoscopic tumor resection have been documented, especially in the CPA. There are still concerns that prevent the endoscope from replacing the microscope, relegating it to an adjunct role (6). Here, we describe our initial experience with fully endoscopic resections of tumors in the CPA performed at a single institution, focusing on surgical techniques, clinical outcomes, and the safety and feasibility of the fully endoscopic technique.

## Methods

### Ethics approval

This study was approved by the research ethics committee of the Affiliated Hospital of Jiangsu University and adhered to relevant rules and guidelines. Informed consent was obtained from all patients.

We retrospectively reviewed 12 consecutive patients harboring CPA lesions who underwent full endoscopic resection from January 2022 to April 2024 at the Department of Neurosurgery at the Hospital of Jiangsu University. During the same period, no surgeries were performed on patients with CPA lesions using only a microscope or endoscope-assisted techniques. Patients without any contradictions to conventional surgery are suitable for fully endoscopic surgery. We gathered all data including age, sex, clinical symptoms, imaging data, Karnofsky Performance Status (KPS) scores before and after surgical treatment, tumor size, pathology, surgical records, and videos, among others. Different symptoms were recorded, and all patients underwent gadolinium-enhanced magnetic resonance imaging (MRI) of the brain, where a tumor, later confirmed by pathology, was diagnosed. All 12 patients underwent full neurologic examinations including detailed cranial nerve findings especially function of the facial nerve and posterior cranial nerves. House–Brackmann (HB) grade was used to evaluate the function of facial nerve postoperation.

### Endoscopic surgery via retrosigmoid approach

All patients underwent fully endoscopic neurosurgery, also known as endoscope-controlled neurosurgery. The procedures were performed under intraoperative electrophysiological monitoring, particularly for the facial nerve in our cases. The fully endoscopic surgeries were performed in a lateral Park-bench position with the head secured in a Mayfield 3-pin head clamp and rotated 15° toward the contralateral side to ensure adequate neck extension. A retrosigmoid straight incision and craniotomy approximately 3 cm × 3 cm in size were performed just inferior to transverse sinuses and medial to the sigmoid sinuses. A short curve in the inferior dura was then made to facilitate the drainage of CSF in the cerebellomedullary cistern under the guidance of a 0° endoscope (4 mm diameter, 18 cm length; Karl Storz, Germany or Mindray, China). Through the combination of gravity assistance and reduced pressure, the corridor between the cerebellum and the petrous bone could be easily opened. After the spontaneous relaxation of the cerebellum, we entered the CPA under the guidance of an endoscope, using retractor surgery technology instead of any fixed brain retractors. Both 0° and 30° endoscopes were used during the surgery, depending on the surgical procedure. In order to prevent fogging of the endoscope, we heated the endoscope by placing it in warm saline before the operation. During the operation, we employed moderate irrigation of the endoscope with warm saline and suction to prevent fogging within the surgical cavity. An endoscope holder (Karl Storz, Tuttlingen, Germany) was used instead of an assistant holder, allowing the surgeon to use both hands for a bi-manual dissection and tumor removal. After exposure was completed, we conducted a preliminary inspection of the surrounding structures, including the regional cranial nerves and vascular anatomy. The facial nerve stimulator assisted the surgeon in detecting the nerve. The possible facial nerve was stimulated, and its response was recorded via an electrophysiological monitor, which was used throughout the surgery to avoid injury to the cranial nerve. Meticulous microneurosurgical techniques were employed throughout the tumor dissection process, including devascularization, debulking, and excision, until total resection of the tumor was achieved. The precise plane between the tumor and healthy neural tissue needed to be carefully identified, for which the endoscope’s approach provided a unique advantage. We routinely opened the internal acoustic canal (IAC) for acoustic neuromas to remove any residual tumor under the guidance of a 30° endoscope. Once the tumor was resected, we stimulated the facial nerve again to confirm its function. After meticulous hemostasis, the dura was re-approximated, the bone flap was replaced, and the scalp was closed in anatomic layers.

### Assessment of the extent of tumor excision

Total resection of meningioma corresponds to Simpson grade I or II resection, while resection of meningioma corresponds to Simpson grade III resection. We also performed routine MRI scans 3 months after surgery in all patients. Those without any residual or diffusion-restricting tumor found were labeled.

## Results

The clinical features of the five male and seven female patients (50–73 years old) are described in **Table 1**. All patients reported diverse onset symptoms. The chief complaints included headache (58.3%), dizziness (41.7%), tinnitus (16.7%), trigeminal paralysis (41.7%), hearing loss (50%), vomiting (8.3%), trigeminal neuralgia (8.3%), and unsteady gait (33.3%). In our study, the mean largest diameter of the lesion was 31.4 mm ± 8.4 mm for acoustic neuromas, 30 mm ± 8.3 mm for meningiomas, and 17.1 mm for a teratoma. Five tumors (41.7%) were larger than 3 cm, and all underwent gross total resection (GTR). Two patients with acoustic neuromas were classified as Koos grade 4, while the remaining three patients were classified as Koos grade 3. Pathological examination confirmed the diagnosis of five acoustic neuromas, six meningiomas, and one teratoma. The extent of resection and clinical outcomes are shown in **Table 2**. One patient suffered transient facial paralysis after surgery (HB grade), which ameliorated during the 3-month follow-up. No direct injury to the pial surface of the brain occurred. All the other patients had resolved clinical symptoms after surgery, with no new onset of neurologic symptoms. No cerebrospinal fluid (CSF) leakage or intracranial infection occurred. All patients were followed up at 1 month, 3 months, and 12 months postoperatively, and then annually. No recurrence or death occurred during follow-up. Taken together, the fully endoscopic retrosigmoid approach provides adequate freedom and safety for the radical resection of tumors.

**Table 1:** Demographics and clinical characteristics of study patients.

Case	Sex	Age (years)	Disease course (days)	Largest diameter (cm)	Clinic symptoms	Pathology
1	F	54	90	3.3	Hearing loss, headache, trigeminal paralysis	Acoustic neuroma
2	F	54	180	3.6	Headache, dizziness, unsteady gait	Meningioma
3	M	66	180	4.2	Headache, dizziness, vomiting, hearing loss, trigeminal paralysis	Meningioma
4	F	57	30	2.3	Hearing loss, dizziness	Acoustic neuroma
5	M	68	4	2.1	Tinnitus, trigeminal paralysis	Meningeoma
6	M	57	730	4.5	Hearing loss, tinnitus, headache, unsteady gait	Acoustic neuroma
7	F	62	7	3.3	Headache, unsteady gait	Meningioma
8	F	50	20	2.8	Hearing loss trigeminal paralysis, headache	Acoustic neuroma
9	F	70	730	2.3	Headache and dizziness	Meningioma
10	F	68	3	2.5	Trigeminal paralysis	Meningioma
11	M	62	270	1.7	Trigeminal neuralgia, dizzy	Teratoma
12	M	73	7	2.8	Hearing loss, unsteady gait	Acoustic neuroma

**Table 2:** Clinical outcome of the patients.

Diagnosis	Acoustic neuroma	Meningioma	Teratoma
Operative time (mean ± SD, min)	269 ± 48.1	181.5 ± 19.1	242
The outcome of clinical symptoms			
Relieved	5	6	1
Invalid	0	0	0
Aggravated	0	0	0
Complications			
Facial paralysis	1	0	0
Dysphagia	0	0	0
KPS mean ± SD			
Before operation	86 ± 5.5	83 ± 5.2	80
Post operation	98 ± 4.5	96.7 ± 5.2	100
Tumor resection extent			
Total resection	5	6	1
Subtotal resection	0	0	0
Partial resection	0	0	0

Total resection of meningioma: Simpson grade I or II. Subtotal resection of meningioma: Simpson grade III.

**Figure 1:**
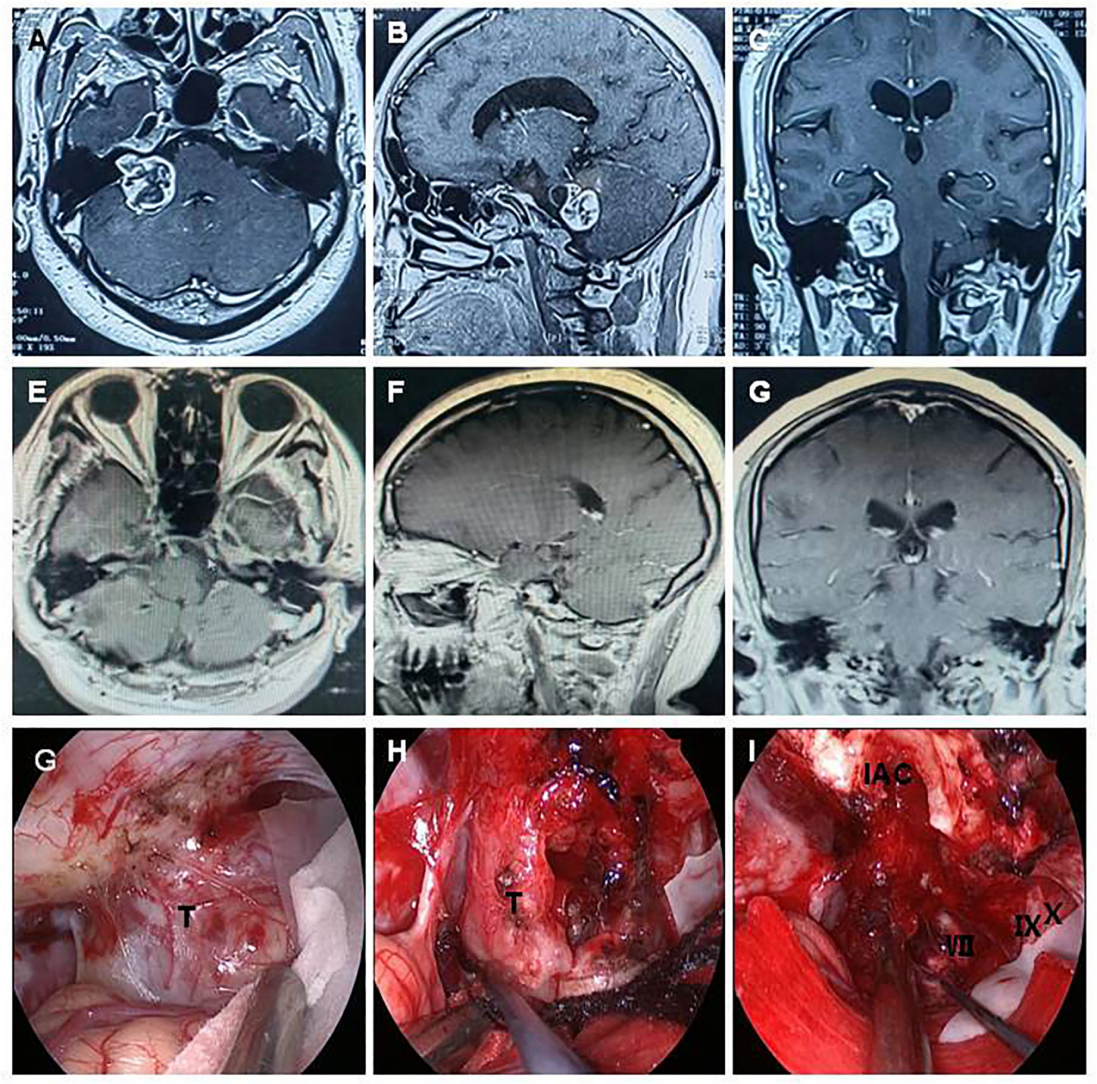
A 54-year-old woman presented with acoustic neuroma. A preoperative MRI scan demonstrated a − 3.3 cm × 2.5 cm lesion located in the CPA region, showing heterogeneous enhancement **(A–C)**. The tumor was completely resected using a fully endoscopic retrosigmoid approach. GTR of the CPA region tumor was confirmed by MRI at 3 months postoperation **(D–F)**. The fully neuroendoscopic surgical procedure demonstrated tumor exposure **(G)**, tumor dissection **(H)**, and complete tumor resection with preservation of the facial nerve **(I)**. T, tumor; VII, facial nerve; IX, glossopharyngeal nerve; X, vagus nerve; IAC, internal acoustic canal.

In the current study, we highlighted three typical cases,with preoperative and postoperative imaging, as well as intraoperative findings. Case 1 involved a 54-year-old woman who had been suffering from hearing decreasing, headache, and trigeminal paralysis for 3 months. A preoperative MRI scan demonstrated a − 3.3 cm × 2.5 cm lesion located in the CPA region, with heterogeneous enhancement (**Figures 1A–C**). The tumor was completely resected through a fully endoscopic retrosigmoid approach. GTR of the CPA region tumor was confirmed by MRI at 3 months postoperation (**Figures 1D–F**). The fully neuroendoscopic surgical procedure exposed the tumor (**Figure 1G**), allowed for its dissection (**Figure 1H**), and resulted in total resection while preserving the facial nerve (**Figure 1I**). Final pathology confirmed the diagnosis of acoustic neuroma. The patient recovered well and was discharged without complications.

**Figure 2:**
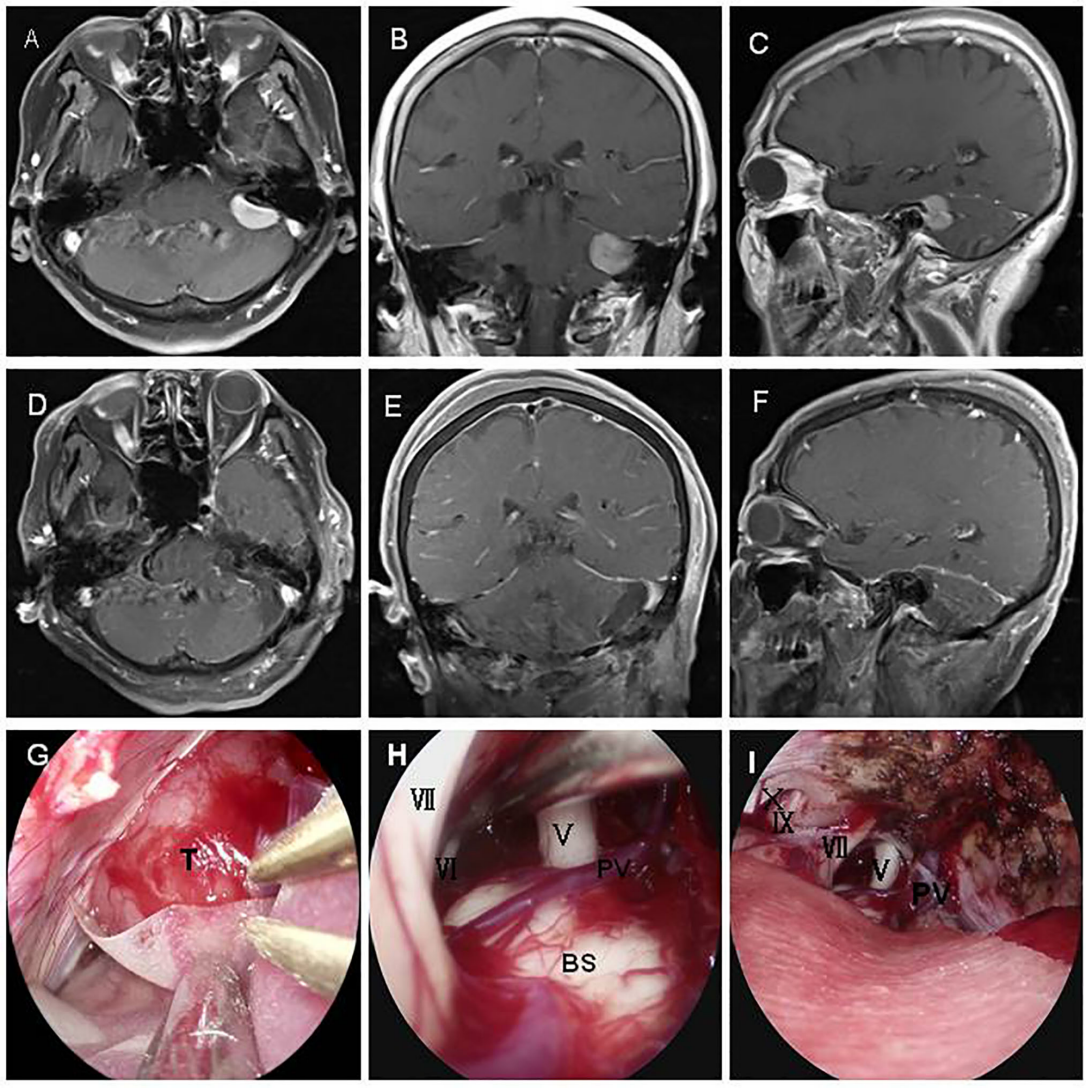
A70-year-old patient presented with a meningioma. A preoperative MRI scan revealed a − 2.2 cm × 1.8 cm lesion in the CPA region with enhancement **(A–C)**. The tumor was completely resected using a fully endoscopic retrosigmoid approach. GTR of the CPA region tumor was confirmed by MR **(D–F)**. A fully neuroendoscopic surgical procedure of the tumor showed exposure of the tumor **(G)**, approaching observation of vital nerves and the brain stem **(H)**, and complete resection of the tumor with preservation of the facial nerve, petrous vein, and posterior group nerves **(I)**. T, tumor; V, trigeminal nerve; VI, abducent nerve; VII,facial nerve; IX, glossopharyngeal nerve; X, vagus nerve; PV, petrosal vein; BS, brain stem.

Case 9 involved a 70-year-old woman whose chief complaints were headache and dizziness for 2 years. Preoperative MRI scan demonstrated a − 2.3 cm × 1.8 cm lesion located in the CPA region with enhancement (**Figures 2A–C**). The tumor was completely resected through a fully endoscopic retrosigmoid approach. GTR of the CPA region tumor was confirmed by MRI (**Figures 2D–F**). A fully neuroendoscopic surgical procedure revealed exposure of the tumor (**Figure 2G**), with detailed observation of vital nerves and the brainstem, among other structures (**Figure 2H**). The tumor was completely resected while preserving the facial nerve, petrous vein, and posterior group nerves (**Figure 2I**). Final pathology confirmed the diagnosis of meningioma. The patient recovered well without any complications at discharge.

**Figure 3:**
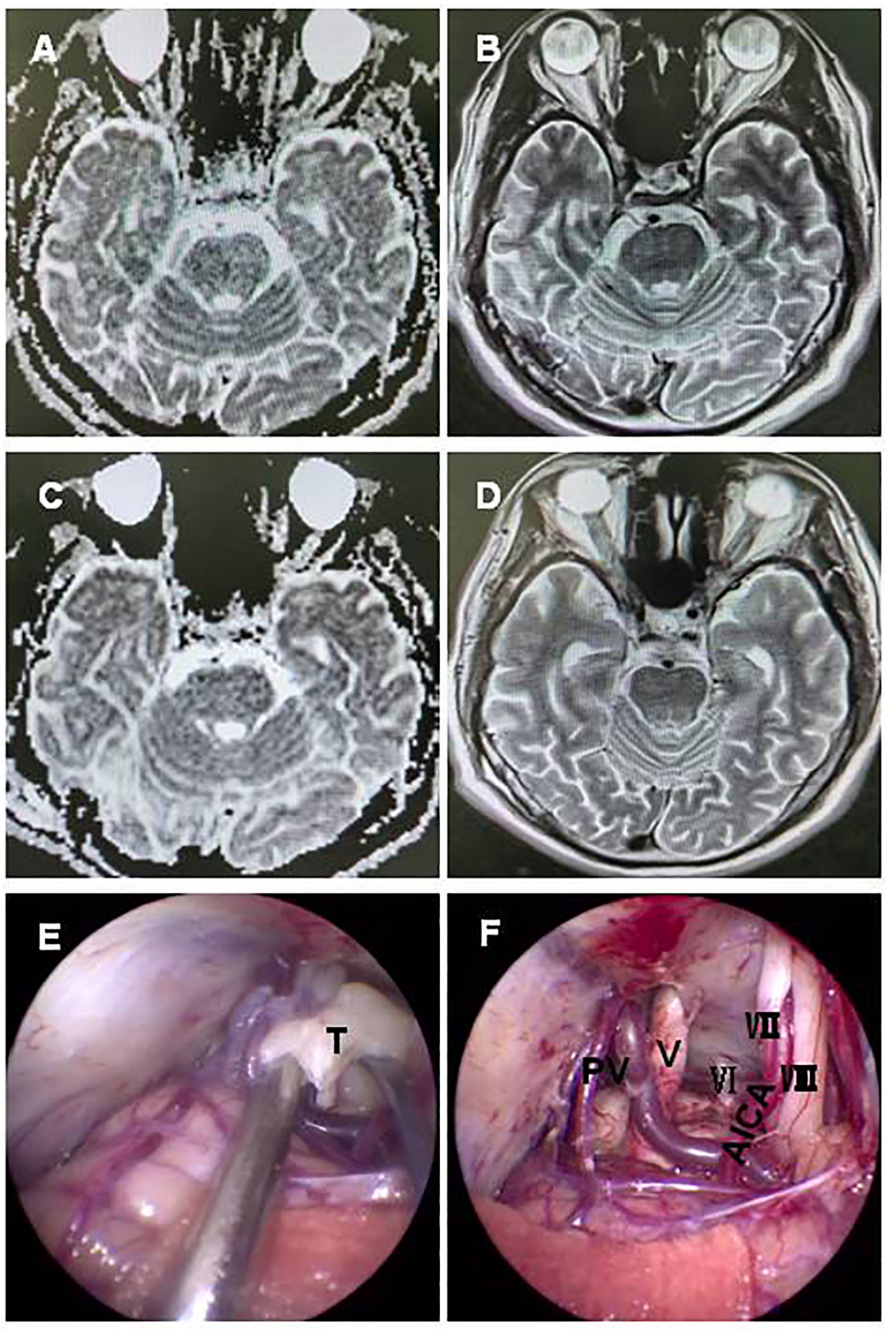
A 62-year-old man with a teratoma. A preoperative MRI scan revealed a − 1.7 cm short T1 signal, long T2 signal lesion located in the CPA region with diffusion restriction **(A, B)**. GTR of the CPA region tumor was confirmed by MRI **(C, D)**. A fully neuroendoscopic surgical procedure of the tumor showed exposure, with the tumor wrapping the petrous vein and trigeminal nerve **(E)**, and complete resection of the tumor with preservation of the facial nerve, petrous vein, and posterior group nerves **(F)**. T, tumor; V, trigeminal nerve; VI, abducent nerve; VII, facial nerve; VIII, auditory nerve; PV, petrosal vein; BS brain stem; AICA, anterior inferior cerebellar artery.

Case 11 involved a 62-year-old man who had been suffering from a serious secondary trigeminal neuralgia and dizziness for approximately 9 months. A preoperative MRI scan demonstrated a − 1.7-cm-long T2 signal lesion located in the CPA region with diffusion restriction (**Figures 3A, B**). The tumor was completely removed via a fully endoscopic approach. GTR of the CPA region tumor was confirmed by MRI (**Figures 3C, D**). The fully endoscopic surgical procedure for the tumor showed exposure of the tumor wrapping the petrous vein and trigeminal nerve (**Figure 3E**), followed by total resection of the tumor and preservation of the facial nerve, petrous vein, and posterior group nerves (**Figure 3F**). Final pathology confirmed teratoma. The patient recovered well and was discharged after 10 days without any complications.

## Discussion

Aggressive surgical resection for CPA tumors, such as vestibular schwannoma, meningioma, and epidermoid, which is a mainstay of management in almost all cases, remains technically challenging due to their deep-seated location and proximity to complex and pivotal neurovascular structure (11–13). Therefore, any refinement in the surgical procedure is desirable. The fully endoscopic technique means the surgeon not only observes the lesion but also operates under the scope of the endoscope, making it is also known as endoscope-controlled surgery. In most previously documented studies, the endoscope only used as an adjunct to the microscope. The experience of fully endoscopic surgery in transcranial procedure is still limited. Here, we introduced the fully endoscopic technique for CPA tumor resection with satisfactory clinical outcomes. This suggests that fully endoscopic surgery is a viable option for tumors in the CPA, with acceptable complications, and may have potential for extensive and routine application.

The endoscope has shed new light on traditional surgery. Unlike the light from a microscope, which enters the surgical field in a conical fashion, an endoscope emits light in a divergent fashion, providing a wide-angled vantage point. This results in a more panoramic view, which is useful for visualizing adjacent structures and allows the neurosurgeon to explore the anatomical landscape lateral and posterior to structures, enabling them to “look around the corner” through approaching observation without unnecessary anatomical manipulation (14). We term conventional surgery, including microscopic keyhole surgery, as an “outdoor surgery mode” because both the microscope and the eye of the surgeon are positioned outside the surgical field. As a result, observation and manipulation are inevitably hindered by the “door” in the surgical corridor, such as the bone window, brain, blood vessels, and nerves. In our experience, the endoscope’s approaching observation can overcome such obstacles and enter the surgical field to provide more precise identification and less invasive manipulation, which we term an “indoor surgery mode”. Given this advantage, even when compared with keyhole surgeries using a microscope, the endoscope offers a more panoramic view and better illumination of deep-seated structures without the need for a fixed retractor, leading to less invasive procedures. Moreover, with the help of a 30° to 45° scope, the surgeon can even see beyond the petrous apex and into the contralateral side.

Recently, the successful application of an endoscope in pituitary adenoma has greatly encouraged neurosurgeons, offering clearer visualization of the surgical field and enabling more total resections (15). The approaching observation and panoramic view provided by endoscopy gained more and more attention from neurosurgeons, ensuring that all surgical procedures are performed under direct vision with fewer blind points, thereby enhancing surgical safety. Some surgeons have started to explore endoscope-assisted neurosurgery to improve illumination during surgery (16, 17). Marchioni et al. argued that an endoscopic support technique should be strongly recommended to avoid residual disease in acoustic neuroma surgery (18). Additionally, there reports documenting the application of the endoscope in microvascular decompression for trigeminal neuralgia and HF, which have shown improved visualization of the CPA neurovasculature compared to the operative microscope (19). However, endoscopy in intracranial surgery is primarily used as an adjunct to conventional microscopic surgical techniques, a practice known as endoscope-assisted microsurgery (20).

In our series, all patients underwent full endoscopic resection without any help from a microscope. Fully endoscopic surgery added no new contraindications of conventional surgery. Twelve tumors were completely resected, and only one patient experienced transient facial paralysis. No hemorrhage, sterile meningitis, or death occurred. The clinical outcomes appear to be superior to those documented for surgery performed with a microscope or endoscope-assisted microscopy. We believe two factors may contribute to the improved outcomes, in addition to the approaching observation and wide-angle panoramic view by the endoscope. The first retractorless technique is widely used in our fully endoscopic neurosurgery, which not only greatly reduces complications associated with cerebellar retraction, such as direct injury to the pial surface of the brain observed in previous traditional microscopic surgeries, but also minimizes damage to bridge veins, particularly the petrosal vein. Additionally, the application of two-handed technology allows the surgeon to freely handle the lesions bimanually with the help of an endoscope holder. Bimanual dexterity under the endoscope enables more confident tumor resection, enduring that nerves and vessels are not damaged, which greatly improves the outcomes. This approach differs significantly from previous reports on the use of endoscopy with a one-handed technique (21).

The main concern with the endoscopic technique is bleeding, which can be difficult to deal with in inexperienced hands. This issue is frequently emphasized by the scientific community when arguing the feasibility of fully endoscopic surgery. In our experience, several techniques contribute to controlling bleeding. First, devascularization is a primary step in preventing bleeding. The approaching observation provided by the endoscope makes it easier for the surgeon to discover and access the feeding artery. Bimanual dexterity under the endoscope offers a significant advantage in dissecting and devascularizing tumors (22). Moderate irrigation with warm saline and the gentle application of appropriately sized gelatin sponges and cottonoids helped control bleeding. Additionally, the two-hand technique helps electrocoagulation using a special bipolar forceps. With enough training, one can manage bleeding freely under the endoscope. Recently, Hong et al. reported their experience managing aneurysms using endoscopic techniques (23).

Endoscopy provides better exposure in the deep-seated regions and allows for finer dissection between tumors, adjacent tissue, and vessels. However, the limitations of fully endoscopic surgery should also be acknowledged. The lack of stereoscopy remains a concern, and therefore, adequate practice and training in the laboratory, along with similar manipulation in this approach, are indispensable. There are also concerns about the possible injury to the blind area posterior to the head of the lens due to the inability to visualize the entry and exit points of instruments when the endoscope is fixed to a holder. To protect this blind area, posterior to the head of the lens, we use a sterilized plastic or cotton pad to cover it, ensuring no damage to those areas. With adequate experience, we can perform fully endoscopic neurosurgery with greater freedom and improved treatment efficacy.

In conclusion, fully endoscopic techniques provide the surgeon with better illumination through a smaller surgical corridor, a wider field of view than that can be accomplished with a microscope, and an improved ability to visualize medial and lateral structures of the CPA without increasing retraction or intracranial bone drilling. With the accumulation of experience and technological advancements, fully endoscopic surgery is safe and feasible for CPA surgery and holds the potential for extensive and routine application by surgeons familiar with endoscopic surgery.

## Data Availability

The raw data supporting the conclusions of this article will be made available by the authors, without undue reservation.

## References

[1] Esser J Walger M Pollet N Klußmann JP Ruge M Goldbrunner R. Vestibular schwannoma: factors in therapy decision-making. Laryngorhinootologie. (2024) 103:176–86. doi: 10.1055/a-2222-0878 38128578

[2] Sampath P Rini D Long DM. Microanatomical variations in the cerebellopontine angle associated with vestibular schwannomas (acoustic neuromas): a retrospective study of 1006 consecutive cases. J Neurosurg. (2000) 92:70–8. doi: 10.3171/jns.2000.92.1.0070 10616085

[3] Verma RP Yadav A Kumar V Ojha BK Chandra A Verma R. Surgical outcomes and predictive factor analysis for facial nerve preservation in patients with cerebellopontine angle (CPA) tumors: A ten-year single institutional study. Cureus. (2024) 16:e61756. doi: 10.7759/cureus.61756 38975511 PMC11226413

[4] Vassilyeva N Mena N Kirov K Diatlova E. Comparative effectiveness of endoscopic and microscopic adenoma removal in acromegaly. Front Endocrinol (Lausanne). (2023) 14:1128345. doi: 10.3389/fendo.2023.1128345 37766690 PMC10519786

[5] Dho YS Kim YH Se YB Han DH Kim JH Park CK. Endoscopic endonasal approach for craniopharyngioma: the importance of the relationship between pituitary stalk and tumor. J Neurosurg. (2018) 129:611–9. doi: 10.3171/2017.4.JNS162143 28960155

[6] Nowak S Matthes M Baldauf J Schroeder HWS. Endoscope-assisted microsurgery for posterior fossa skull base meningioma surgery: technique and results. Oper Neurosurg (Hagerstown). (2024) 27(2):194–204. doi: 10.1227/ons.0000000000001093 38385687

[7] El Beltagy MA Atteya MME. Benefits of endoscope-assisted microsurgery in the management of pediatric brain tumors. Neurosurg Focus. (2021) 50:E7. doi: 10.3171/2020.10.FOCUS20620 33386008

[8] Yang D Shu W Du T Li J Zhu H. Safety and efficacy of endoscope-assisted versus microscopic microvascular decompression surgery for hemifacial spasm: a prospective cohort study. Acta Neurol Belg. (2024) 124(5):1555–60. doi: 10.1007/s13760-024-02539-4 38625498

[9] Naimi B Duffy A Garvey E Urdang Z Farquhar D Kelly P. Trends in endoscopic and microscopic approaches to transsphenoidal pituitary surgery in the US. Laryngoscope. (2023) 133:2135–40. doi: 10.1002/lary.v133.9 37318105

[10] Cai Q Li Z Guo Q Wang W Ji B Chen Z. Microvascular decompression using a fully transcranial neuroendoscopic approach. Br J Neurosurg. (2023) 37:1375–8. doi: 10.1080/02688697.2020.1820943 33491507

[11] Dunn IF Bi WL Mukundan S Delman BN Parish J Atkins T. Olson JJ.Congress of neurological surgeons systematic review and evidence-based guidelines on the role of imaging in the diagnosis and management of patients with vestibular schwannomas. Neurosurgery. (2018) 82:E32–4. doi: 10.1093/neuros/nyx510 29309686

[12] Singh R Prasad RS Singh A. Evaluation of cerebellopontine angle epidermoid presenting with cranial nerve deficit: A surgical perspective. Asian J Neurosurg. (2020) 15:573–8. doi: 10.4103/ajns.AJNS_226_20 PMC7591210 33145209

[13] Ali MS Magill ST McDermott MW. Petrous face meningiomas. Handb Clin Neurol. (2020) 170:157–65. doi: 10.1016/B978-0-12-822198-3.00037-9 32586487

[14] Tariq F Jumah F Ravipati K Ortiz-Torres M Carr SB Chicoine MR. Advances in cranial surgery. Mo Med. (2024) 121:136–41. PMC11057866 38694609

[15] Guinto G Guinto-Nishimura GY Sangrador-Deitos MV Uribe-Pacheco R Soto-Martinez R Gallardo D. Current and future perspectives of microscopic and endoscopic transsphenoidal surgery for pituitary adenomas: A narrative review. Arch Med Res. (2023) 54:102872. doi: 10.1016/j.arcmed.2023.102872 37633807

[16] Fouda MA Jeelani Y Gokoglu A Iyer RR Cohen AR. Endoscope-assisted microsurgical retrosigmoid approach to the lateral posterior fossa: Cadaveric model and a review of literature. Surg Neurol Int. (2021) 12:416. doi: 10.25259/SNI_157_2021 34513180 PMC8422411

[17] Oertel J Fischer G Linsler S Huelser M Sippl C Teping F. Endoscope-assisted resection of brainstem cavernous malformations. Neurosurg Rev. (2022) 45:2823–36. doi: 10.1007/s10143-022-01793-5 PMC9349151 35499666

[18] Marchioni D Gazzini L Boaria F Pinna G Masotto B Rubini A. Is endoscopic inspection necessary to detect residual disease in acoustic neuroma surgery? Eur Arch Otorhinolaryngol. (2019) 276:2155–63. doi: 10.1007/s00405-019-05442-4 31028535

[19] Chen L Shang Y Zhang Y Zhao Y. Endoscopic microvascular decompression versus microscopic microvascular decompression for trigeminal neuralgia: A systematic review and meta-analysis. J Clin Neurosci. (2023) 117:73–8. doi: 10.1016/j.jocn.2023.09.009 37776679

[20] Abolfotoh M Bi WL Hong CK Almefty KK Boskovitz A Dunn IF. The combined microscopic-endoscopic technique for radical resection of cerebellopontine angle tumors. J Neurosurg. (2015) 123:1301–11. doi: 10.3171/2014.10.JNS141465 25909571

[21] Marchioni D Carner M Rubini A Nogueira JF Masotto B Alicandri-Ciufelli M. The fully endoscopic acoustic neuroma surgery. Otolaryngol Clin N Am. (2016) 49:1227–36. FEBORL-HNSd. doi: 10.1016/j.otc.2016.05.014 27565388

[22] Spazzapan P Velnar T Bosnjak R. Endoscopic supra cerebellar infratentorial approach to pineal and pos terior third ventricle lesions in prone position with head extension: a technical note. Neurol Res. (2020) 42:1070–3. doi: 10.1080/01616412.2020.1805926 32892737

[23] Xiao LM Tang B Xie SH Huang GL Wang ZG Zeng EM. Endoscopic endonasal clipping of anterior circulation aneurysm: surgical techniques and results. World Neurosurg. (2018) 115:e33–44. doi: 10.1016/j.wneu.2018.03.093 29574221

